# Chinese herbal medicine for premature ovarian insufficiency: A protocol for systematic review

**DOI:** 10.1097/MD.0000000000019371

**Published:** 2020-03-13

**Authors:** Yifeng Shao, Huihao Zhou, Meng Zhou, Pian Ying, Zhitao Yao, Xuelu Jiang

**Affiliations:** a First College of Clinical Medicine, Zhejiang Chinese Medical University; b Department of Gynecology, First Affiliated Hospital of Zhejiang Chinese Medical University, Hangzhou, China.

**Keywords:** premature ovarian insufficiency, Chinese herbal medicine, meta-analysis, systematic review, protocol

## Abstract

**Background:**

Premature ovarian insufficiency (POI) is the loss of function of the ovaries before age 40. Chinese herbal medicine (CHM) has been treating POI for long time. Therefore, we conduct this study to assess the efficacy and safety of CHM for POI.

**Methods:**

Seven databases will be searched from inception to December 31, 2018: PubMed, Embase, Cochrane Library, Chinese National Knowledge Infrastructure (CNKI), China Biology Medicine disc (CBM), WanFang Database, and Chongqing Chinese Scientific Journal Database (CQVIP). Randomized controlled trials that used CHM will be included. Two reviewers will independently complete the study selection, data extraction, and study quality assessment according to Cochrane Collaboration. All the data will be analyzed using Review Manage 5.3 software.

**Results:**

This study will generate a comprehensive summary on effectiveness and safety of CHM for POI.

**Conclusion:**

This study may be beneficial to health policymakers, clinicians, and patients with regard to the use of CHM in POI treatment.

**Trial registration number:**

PROSPERO CRD 42019144629.

## 1 Introduction

Premature ovarian insufficiency (POI) is defined as the cessation of function of the ovaries before the age of 40 years.^[1]^ It is known as premature ovarian failure or primary ovarian insufficiency before.^[2]^ Although it has been estimated that POI affects about 1% of the female population, the prevalence is less certain.^[3]^ The etiology in most cases is usually unclear though some potential causes such as genetic, autoimmune, iatrogenic, environmental factors play a role in POI.^[4]^ Health complications of POI include menopausal symptoms, menstrual disturbance, infertility, and psychosocial issues.^[5]^

Most health complications of POI are directly related to ovarian hormone deficiency.^[6]^ Hormone replacement therapy (HRT) plays a seminal role in treatment of POI.^[7]^ However, it shows increased health risks related to the use of HRT, including breast cancer, stroke, and cardiovascular disease.^[8]^ Therefore, many patients are turning to complementary and alternative medicine (CAM) for relief of symptoms of POI. In all CAM, traditional Chinese medicine (TCM) has become more popular in recent year.^[9]^ Chinese herbal medicine (CHM), one of the commonly therapeutic approaches of TCM, has been widely used for the treatment of POI in China and other Asian countries.^[10]^

Many clinical studies report that CHM had a positive effect in treating women with POI and it is traditionally regarded as having few side effects.^[9]^ However, most of the clinical trials provided insufficient evidence due to the small sample sizes. Systematic review is a type of literature review that uses systematic methods to collect data, critically appraise research studies, and synthesize studies. Therefore, we will conduct the present systematic review to evaluate the therapeutic effects and safety of CHM for the treatment of POI.

## 2 Methods

### 2.1 Study registration

The protocol has been registered on PROSPERO 2019 (registration number: CRD 42019144629). This protocol will be reported according to the Preferred Reporting Items for Systematic Reviews and Meta-Analyses Protocols (PRISMA-P) statement guidelines.

### 2.2 Data source

We will conduct the comprehensive search in following databases: PubMed, Embase, Cochrane Library, Chinese National Knowledge Infrastructure (CNKI), China Biology Medicine disc (CBM), WanFang Database, and Chongqing Chinese Scientific Journal Database (CQVIP). These databases will be searched from inception to December 31, 2018. No restriction on language was applied. The search words will be used as follows: (((“herb” OR “herbal medicine” OR “Chinese herb” OR “Chinese herbal medicine” OR “traditional medicine” OR “traditional Chinese medicine”) AND (“premature ovarian insufficiency” OR “primary ovarian insufficiency” OR “premature ovarian failure”)) AND (“random” OR “randomized” OR “controlled trial” OR “clinical trial” OR “randomized controlled trial”)). The search words will be modified to adapt to different databases. Furthermore, potentially relevant studies will be identified by manual search of reference list of included trials and review articles.

### 2.3 Study Selection

#### 2.3.1 Type of studies

All randomized controlled trials (RCTs) with concrete random sequence generation method will be selected. Retrospective studies, cohort studies, qualitative studies, case reports or series, experience summaries, and animal experiments were excluded. There is no restriction on language.

#### 2.3.2 Type of participants

Patients enrolled is diagnosed as POI according to “The ESHRE Guideline Group on POI.”^[11]^ Patients should meet all of the following criteria: women younger than 40 years old, amenorrhea or oligoamenorrhea for at least 4 months; an elevated serum follicle-stimulating hormone (FSH) levels (>25 IU/L) on at least 2 separate occasions apart (>4 weeks).

#### 2.3.3 Type of interventions

The intervention group is treated with CHM therapy or combined with HRT. The CHM therapy can be in any dose or administrated methods. The control group is subject to HRT alone or placebo. Study comparing with other TCM therapy (such as acupuncture and moxibustion) will be excluded.

#### 2.3.4 Type of outcome measures

Primary outcomes including: Secondary outcomes including: hormone levels of E2, FSH, and LH after treatment; adverse events.

### 2.4 Data collection and analysis

#### 2.4.1 Data extraction and management

All included studies will be read by 2 independent authors who extract the data by using a predesigned table. The data extraction items include: 1st author, year of publication, country, sample size, baseline condition of participants, intervention and comparison, treatment duration, outcome, adverse effects, and follow-up. Disagreements will be resolved by mutual discussion.

#### 2.4.2 Assessment of the risk of bias in the included studies

The 2 authors independently perform a methodologic quality assessment of the included studies, which is based on the Cochrane Collaboration's tool for assessing risk of bias (ROB). Seven components were evaluated: random sequence generation, allocation concealment, blinding of participants and personnel, blinding of outcome assessment, incomplete outcome data, selective reporting and other bias. The ROB for each item was classified as low, unclear, and high. Any discrepancies were discussed with a further author.

#### 2.4.3 Measurement of treatment effect

Dichotomous data will be presented as relative risk with 95% confidence interval (CI) and continuous data were reported as weighted mean difference or standardized mean difference with 95% CI.

#### 2.4.4 Dealing with missing data

If data are missing or data are expressed as diagrams without absolute numbers, the authors of the included studies will be contacted by phone, fax, or email to obtain missing information. All analyses will be performed according to intent-to-treat principle. The study will be excluded if we fail to obtain the missing data.

#### 2.4.5 Assessment of heterogeneity

Statistical heterogeneity of the pooled results will be assessed using Cochrane *Q* test and *I*^2^ test. The random-effects model is applied when significant heterogeneity existed (*P* < .05 or *I*^2^ > 50%); otherwise, the fixed-effects model will be adopted. If heterogeneity is detected, we will conduct subgroup analyses or sensitivity analyses.

#### 2.4.6 Assessment of publication biases

Funnel plot will be used to detect publication bias if there are more than 10 eligible studies. If funnel plot asymmetry is discovered, we will attempt to distinguish the potential source of the asymmetry.

#### 2.4.7 Data synthesis

Review Manage (RevMan), Version 5.3.5 software (The Nordic Cochrane Centre, Copenhagen, Denmark) is used for data synthesis. RCTs will be synthesized according to the type of intervention and control as follows: CHM group vs HRT control; CHM vs placebo; CHM plus HRT group vs HRT control. If there is obvious clinical heterogeneity, and inability to find out the source of heterogeneity, we will conduct descriptive analysis.

#### 2.4.8 Subgroup analysis

If there are sufficient studies to investigate the source of heterogeneity, we will conduct subgroup analysis. Subgroup analysis criteria will include course of disease, treatment period, type of herbal medicine, and dosage.

#### 2.4.9 Sensitivity analysis

We will carry out sensitivity analysis for the review's primary outcomes to explore the robustness of the results. It will be performed according to the following criteria: high levels of missing data, sample size, and methodologic quality.

#### 2.4.10 Quality of evidence

The overall quality of this study will be evaluated by using the Grading of Recommendations Assessment, Development and Evaluation (GRADE). The level of evidence will be categorized as high, moderate, low, and very low.

### 2.5 Ethics and dissemination

Ethical approval is not required because this protocol is for a systematic review. This study will be published in a peer-reviewed journal and disseminated electronically and in print.

## 3 Discussion

The POI comprises symptoms of decline on ovarian function before 40 years old. CHM treatment has become more popular due to deficiency of conventional therapies. However, it lacks available evidence for clinical use of CHM for POI. This systematic review will conducted a comprehensive synthesis to evaluate effectiveness and safety of CHM for POI. We hope that this review can provide a clinical evidence for treating POI, which is beneficial to patients and practitioners.

## Author contributions

**Data curation:** Yifeng Shao, Meng Zhou.

**Formal analysis:** Yifeng Shao, Huihao Zhou.

**Methodology:** Xuelu Jiang.

**Resources:** Yifeng Shao, Huihao Zhou, Pian Ying.

**Software:** Yifeng Shao, Huihao Zhou, Meng Zhou.

**Supervision:** Zhitao Yao, Xuelu Jiang.

**Writing – original draft:** Yifeng Shao.

**Writing – review & editing:** Yifeng Shao, Zhitao Yao.
